# Room-temperature photo-induced martensitic transformation in a protein crystal. Corrigendum

**DOI:** 10.1107/S2052252519007851

**Published:** 2019-06-06

**Authors:** Steven Dajnowicz, Patricia S. Langan, Kevin L. Weiss, Ilia N. Ivanov, Andrey Kovalevsky

**Affiliations:** a Department of Chemistry and Biochemistry, University of Toledo, Toledo, Ohio 43606, USA; b Neutron Scattering Division, Oak Ridge National Laboratory, Oak Ridge, Tennessee 37831, USA; c Center for Nanophase Materials Sciences, Oak Ridge National Laboratory, Oak Ridge, Tennessee 37831, USA

**Keywords:** martensitic transformations, reversibly switchable fluorescent proteins, fluorescence, tetramerization, isomerization, chromophore deprotonation, UV–vis absorption spectroscopy, room-temperature X-ray photocrystallography

## Abstract

The article by Dajnowicz *et al.* [*IUCrJ* (2019). **6**, 619–629] is withdrawn.

## Abstract 2

Corrigendum to the article by Dajnowicz *et al.* [*IUCrJ* (2019). **6**, 619–629].

Since acceptance for publication, the authors of Dajnowicz *et al.* (2019 ▸) have become aware that, while the data presented regarding the crystal structures, UV–Vis and fluorescence spectra are correct, the authors cannot conclude with certainty that the photo-induced structural changes can occur *in crystallo*, *i.e.* as a martensitic transformation. The article has therefore been withdrawn.
